# Predicting the Impact of Glycosylation on the Structure and Thermostability of *Helicobacter pylori* Blood Group Binding Adhesin

**DOI:** 10.3390/biom15101480

**Published:** 2025-10-21

**Authors:** Daniel Sijmons, Heber Islas Rios, Benjamin R. Turner, Emma Wanicek, Jessica K. Holien, Anna K. Walduck, Paul A. Ramsland

**Affiliations:** 1 School of Science, RMIT University, Melbourne, VIC 3000, Australia; daniel.sijmons@unimelb.edu.au (D.S.); s4063707@student.rmit.edu.au (H.I.R.); ben.turner@student.unimelb.edu.au (B.R.T.); emma.wanicek3@rmit.edu.au (E.W.); jessica.holien@rmit.edu.au (J.K.H.); anwalduck@csu.edu.au (A.K.W.); 2 Department of Microbiology and Immunology, Peter Doherty Institute for Infection and Immunity, University of Melbourne, Melbourne, VIC 3000, Australia; 3 Rural Health Research Institute, Charles Sturt University, Orange, NSW 2800, Australia; 4 Department of Immunology, Monash University, Melbourne, VIC 3004, Australia; 5 Department of Surgery, Austin Health, The University of Melbourne, Heidelberg, VIC 3084, Australia

**Keywords:** glycosylation, protein stability, bacterial adhesion proteins, protein folding

## Abstract

Post-translational modifications (PTMs) are critically important for protein structure and function, with glycosylation being one of the most common forms of PTM. The gastric pathogen *Helicobacter pylori* has a general glycosylation system, which performs complex glycosylation of lipopolysaccharide, flagella proteins, and outer membrane proteins (OMPs). One of the best-described OMPs of *H. pylori* is the blood group binding adhesin (BabA), which interacts with the Lewis histo-blood group antigen, Lewis b. The 3D structure for BabA has been determined, and the ligand specifically described. Although BabA is reported to be a glycoprotein, there are limited data examining the effects of glycosylation on the structure and function of this protein. This study examined the folding and thermostability of non-glycosylated recombinant BabA and used computational approaches to predict the effect of glycosylation on the protein, with a focus on its possible heterologous expression in mammalian cells. Three potential O-linked and three potential N-linked glycosylation sites were predicted. Furthermore, the effect of glycan shielding on the solvent-accessible surface area of BabA was examined. Molecular dynamics simulations highlighted local indicators, including root mean square fluctuation and the number of protein-glycan contacts that were affected by glycosylation. Taken together, the findings support a role of glycans in surface shielding and promoting local stabilization in specific areas of the BabA protein. This study helps to strengthen the understanding of the importance of glycosylation and the role it plays in the structure, function, and stability of *H. pylori* proteins.

## 1. Introduction

Protein glycosylation is an important post-translational modification (PTM) that heavily influences many molecular and cellular processes and has been found to influence protein structure and function [1]. The important gastric pathogen, *Helicobacter pylori*, is part of a group of microbes reported to perform complex glycosylation, and the closely related *Campylobacter jejuni* was the first bacterium to have glycosylation identified in cellular processes [2,3]. *H. pylori* employs an array of outer membrane proteins (OMPs) which allow it to attach to the host gastric mucosa and gastric epithelial cells. One of the most studied proteins in the adhesion of *H. pylori* is the blood group binding adhesin (BabA) [4,5,6,7,8]. BabA can exploit the glycan expression of human cells by targeting ligands such as the histo-blood group antigen Lewis b (Le^b^) and the H1 antigen of the O-type blood group [6,8,9,10,11]. There have been several studies focusing on the structural biology of BabA, and several crystal structures of the outer membrane region have been deposited in the Protein Data Bank (PDB) [4,12]. All the available BabA structures were determined for non-glycosylated versions, which means that there is no experimental 3D structure currently available of the protein in a glycosylated form.

When expressed in prokaryotic systems such as *E. coli*, BabA is a relatively low-expressing protein; therefore, a previous study employed nanobodies to stabilize the protein and assist in crystal formation [12]. The system used by *H. pylori* to glycosylate outer membrane proteins including BabA has been recently described and involves several ligation enzymes such as WecA, Wzk, and WaaL, which are also involved in lipopolysaccharide (LPS) biosynthesis. It was reported that the sequential process of glycosylation results in a two-step molecular weight upshift pattern as the proteins are moved from the cytoplasm to the inner membrane, and subsequently to the outer membrane [5]. Knockout of glycosylation enzymes caused an impairment of protein structural stability, protease resistance, and binding ability in *H. pylori* OMPs, including BabA, as well as impairment of the LPS formation [5]. Additionally, it has been recently shown through gene knockout and antibody targeting of glycotransferases that the depletion of the glycosylation of *H. pylori* OMPs results in a notable reduction in the molecular weight of these proteins, including BabA, and a reduction in the overall adhesion of the bacterial cells [13]. While BabA was identified as a putative glycoprotein, there has not yet been any direct characterization of the glycans (N- or O-linked) by mass spectrometry. In addition, there has been some recent discussion that the apparent two-step gel shift observed for OMPs may not directly confirm protein glycosylation in *H. pylori* [14]. Thus, further investigation into glycosylation in *H. pylori* is warranted.

Only a few glycosylated proteins have been previously described in *H. pylori*, with initial studies focusing primarily on the synthesis of flagellar proteins, FlaA and FlaB, which both have been confirmed to be modified with O-linked pseudaminic acids [15,16]. In contrast, there has been extensive research into bacterial glycosylation in *C. jejuni* [5,17,18,19,20,21,22]. While *H. pylori* BabA has been extensively studied, research on its glycosylation and its impact on protein stability and function has been limited [5,6,7,10,12,23]. Studies focused on other proteins have suggested that glycosylation can have significant impacts on structure and function, and molecular dynamics (MD) simulations of selected proteins in the Protein Data Bank (PDB) reveal that N-glycosylated proteins tend to display lower mobility compared to the non-glycosylated versions [24]. Another study demonstrated that proteins displayed a preference for more open, flexible, and diverse conformations in the presence of glycosylation compared to the same non-glycosylated proteins [25].

We postulated that altering glycosylation on the surface of the protein would alter the physical properties of the protein, such as thermal stability, available surface area and flexibility. This study initially examined the thermostability of recombinant BabA produced in the *E. coli* expression system. After observing the melting temperature of the non-glycosylated BabA to be unexpectedly low, our investigation moved toward predicting the potential glycosylation of the BabA extracellular region. Our main motivation was to predict the effect of glycosylation on BabA if it were recombinantly expressed in a mammalian cell expression system. Three accessible O-linked glycosylation sites were computationally modelled, and two were assessed due to a relatively low solvent accessible surface area (SASA) score for the third, and three accessible N-linked glycosylation sites were computationally modelled to predict how the glycans can shield the surface of the BabA protein, possibly acting to increase thermal stability in glycosylated BabA. These findings suggest that production of BabA in an expression system that glycosylates the protein is needed to fully elucidate glycan recognition by BabA and to facilitate future drug development efforts.

## 2. Methodology

### 2.1. BabA Expression and Purification

To recombinantly express BabA, BabA residues 18−501 were cloned into the pET-22b(+) periplasmic expression vector. These corresponded to the same residues used by Hage et al. [6], which represents the extracellular portion of the BabA. The carboxyl-terminal β-barrel portion of the BabA that is embedded in the outer membrane was not included in the construct to facilitate soluble expression. Codon optimization of the sequence for *E. coli* expression and cloning was completed by GenScript. The plasmid was transformed into competent *E. coli* BL21 Star^TM^ (DE3) cells (Thermo Fisher, Waltham, MA, USA), plated on LB agar with 100 μg/mL ampicillin for 24 h at 37 °C. The colonies were selected and added to LB broth and grown at 37 °C to an optical density (OD) of 0.6 (600 nm), and cultures were induced with 0.5 mM isopropyl β-D-1-thiogalactopyranoside (IPTG) at 16 °C for 16 h in an orbital shaker incubator at 200 rpm. Following expression, periplasmic extraction was performed using a modification of the protocol described by Hernandez Rollan et al. [26]. Following incubation at 16 °C, the culture was centrifuged at 8000× *g* at 4 °C for 20 min and the supernatant was discarded. The pellet was then resuspended in a buffer comprising 200 mM Tris-HCl pH 8, 500 mM sucrose, and 1 mM EDTA at a volume of 3 mL per gram of cells. The suspension was then incubated at room temperature for 10 min and cold-shocked by the addition of 3 mL of ice-cold MilliQ water per gram of cells and then incubated on ice for a further 10 min. The solution was then centrifuged at 8000× *g* for 20 min and the periplasmic extract was collected and stored at 4 °C for subsequent purification. The sample was purified on a HisTrap FF 1 mL nickel column (Cytiva, Waltham, MA, USA) using immobilized metal affinity chromatography (IMAC) on the Äkta Pure chromatography system (Cytiva, USA). For purification, a binding buffer of 20 mM sodium phosphate, 0.5 M NaCl, 20 mM imidazole, pH 7.4, and an elution buffer of 20 mM sodium phosphate, 0.5 M NaCl, 500 mM imidazole, pH 7.4 were used. The column was washed with 5 column volumes (CV) of MilliQ water and then equilibrated with 5 CV binding buffer (20 mM sodium phosphate, 0.5 M NaCl, 20 mM imidazole, pH 7.4). Forty mL of periplasmic fraction was loaded onto the column at 1 mL/minute. The column was washed with 10 CV binding buffer. BabA was eluted off the column over a gradient (20–500 mM imidazole) over 5 CV, with the protein observed to elute at approximately 150 mM imidazole. Expression and purity were then verified by sodium dodecyl sulfate polyacrylamide gel electrophoresis (SDS-PAGE; BioRad Laboratories, Hercules, CA, USA) and expression was confirmed by western blot (Figure S1).

### 2.2. Solution Property Assessment of BabA by Dynamic Light Scattering

The solution properties of purified recombinant BabA protein were assessed by dynamic light scattering (DLS). DLS measurements were performed on the Zetasizer Nano ZS (Malvern Instruments, Malvern, UK), using a 633 nm laser and a single detector located at 173°. Prior to DLS, purified BabA was centrifuged at 15,000 rpm at room temperature for 10 min. The supernatant was transferred to low-volume cuvettes and allowed to equilibrate at room temperature for 30 min prior to obtaining DLS data. Five measurements were performed at 25° C at various storage timepoints using automated data acquisition parameters. The z-average hydrodynamic diameter (*D_H_*) and polydispersity index (PDI) were determined by the cumulants method, and all data were analyzed using the Zetasizer software 8.02 (Malvern Instruments, UK). The mean and standard deviation were calculated from the five measurements. Graphs were produced using Microsoft Excel 365 and GraphPad Prism 10.2.3 (Dotmatics, Woburn, MA, USA).

### 2.3. Thermal Stability of Recombinant BabA

The thermostability of recombinant BabA was examined using nano differential scanning fluorometry (nanoDSF) to measure the protein thermal denaturation (Tycho, NanoTemper Technologies GmbH, München, Germany). Samples were loaded in nanoDSF-grade standard capillaries (NanoTemper Technologies GmbH, München, Germany) [27] and exposed to thermal stress via the ramping of temperature from 35 °C to 95 °C at a rate of 30 °C per minute. Fluorescence was measured at 350 nm and 330 nm, and the fluorescence ratio was calculated between 35 °C and 95 °C. The first derivative of the 350 nm/330 nm fluorescence ratio was used to determine the melting point of the BabA protein.

Circular dichroism (CD) spectroscopy was used to assess the secondary structure of recombinant BabA at 0.15 mg/mL in 0.5× PBS. Spectrosil quartz 1 mm pathlength cuvettes (Starna Scientific Ltd., Ilford, UK) containing 300 μL of protein solution were loaded into a Jasco J-1500 CD Spectrometer (Jasco Inc., Easton, MD, USA). Spectra were collected between 180 and 260 nm wavelengths at 20° C with a data pitch of 0.1 nm, a scan speed of 20 nm/min, and five accumulations. The thermal unfolding of BabA was then determined by CD with spectra obtained from a 0.2 mg/mL BabA solution in 0.75× PBS every 2.5 °C degrees between 25 °C and 60 °C. Scans were acquired between 195 and 250 nm with a data pitch of 1 nm, a scan speed of 50 nm/min, and five accumulations at each temperature point. The secondary structure composition from CD scans was determined using two online tools: DichroWeb [28] and the single-spectrum analysis and fold recognition program from BeStSel [29]. The output data from both programs were collated in Microsoft Excel before analysis and graphing using RStudio (R version 4.3.1) and GraphPad Prism (version 10.2.3).

### 2.4. Glycosylation Prediction

The BabA sequence was derived from the crystal structure PDB: 4ZH7 [6]. Potential glycosylation sites were identified utilizing NetNGlyc 1.0 [30] and NetOGlyc 4.0 [31,32] bioinformatics software and the output data were processed using GraphPad prism 10.2.3 and Microsoft Excel. The cutoff score for glycosylation prediction for both N- and O-glycosylation tools was set at 0.5. Additionally, GLYCAM-web was used to glycosylate predicted sites, using the inbuilt oligosaccharide library to add two types of N-glycans: the high mannose core and complex type, as well as the O-core type 2 at the O-glycosylation site, as identified by GLYCAM-web [33,34]. The sites predicted by GLYCAM-Web were determined by the solvent-accessible surface area (SASA) score to rank the potential N-linked and O-linked glycosylation sites. 3D structures of the glycosylated protein were downloaded and viewed in Discovery studio, and the images generated were annotated in Adobe Photoshop 2022. The BabA 3D model was glycosylated at the sites predicted for N-glycosylation and O-glycosylation by GLYCAM-web [33], specifically at residues Asn173, Asn275, and Asn314 for N-glycosylation sites, and at Thr397 and Thr400 for O-glycosylation sites. To evaluate the structural impact of glycosylation, four systems were generated: System 1 corresponds to the non-glycosylated BabA model and was used as a control; System 2 includes Core N-glycans, specifically Manα1-3(Manα1-6)Manβ1-4GlcNAcβ1-4GlcNAcβ1-Asn at the predicted N-glycosylation sites. System 3 features complex-type N-glycans with core fucosylation, namely Neu5Acα2-6Galβ1-4GlcNAcβ1-2Manα1-3(Neu5Acα2-6Galβ1-4GlcNAcβ1-2Manα1-6)Manβ1-4GlcNAcβ1-4(Fucα1-6)GlcNAcβ1–Asn. System 4 combines the same complex-type N-glycans as in System 3 with additional Core 2 O-glycans Galβ1-3(GlcNAcβ1-6)GalNAcα1-Thr at Thr397 and Thr400 (Table S1).

### 2.5. Analysis of Shielding Effects of Glycosylation of BabA Using GlycoSHIELD

The BabA 3D model was examined on the webserver GlycoSHIELD (www.glycoshield.eu, accessed on 19 April 2024), to rapidly explore the potential shielding effect of glycosylation on the protein [34,35]. GlycoSHIELD contains a library of 68 glycans and 30,000 conformers per glycan. Glycans are grafted onto selected glycosylation sites on the protein. GlycoSHIELD then aligns every glycan conformer with the protein backbone via a tripeptide anchor onto matching residues on the protein. This ensemble of glycans is then tested for clashes with protein atoms within a soft threshold radius of 0.7 Å and 3.5 Å, taking the dynamic nature of amino acid lateral chains on the protein surface into account and excluding any clashing conformers [35].

### 2.6. Molecular Dynamics Simulations

Molecular dynamics (MD) simulations were performed using GROMACS 2024 [36]. The simulation parameters were prepared using CHARMM-GUI (http://www.charmm-gui.org, accessed on 22 September 2025) [37,38,39]. For glycosylation, the glycans were also built and covalently attached using CHARMM-GUI and are described in Table S1 [40,41,42]. Each MD simulation was run for 200 ns in total, with a 5 ns equilibration time and the Nose-Hoover thermostat set at a temperature of 303.15 K [43] and at 1 bar using the Parrinello-Rahman barostat [44]. The force field selection consisted of FF19SB for the protein, GLYCAM06j for the glycans, and OPC for the water model. The simulations were run using CHARMM-GUI’s standard input settings [39,45]. All the systems were simulated in duplicate, and additional simulations were run using an alternative water model, namely TIP3P. After producing 200 ns of MD simulations for the four BabA systems, the results were analyzed considering both global and local structural changes. To assess global protein stability, the following global indicators were used: root mean square deviation (RMSD), radius of gyration, and the number of hydrogen bonds (Hbonds). For local structural changes, the analysis focused on root mean square fluctuation (RMSF) and the number of contacts between the glycans and the protein. For these indicators, the entire protein was considered, except for RMSD and RMSF, which were calculated using the protein backbone. Particular attention was given to the glycosylated regions and the Le^b^ binding site, as well as any other regions showing differences in flexibility across the four systems evaluated. All indicators were computed using GROMACS tools, and data analysis, as well as graph generation, were performed using Python, while trajectory inspection and image generation were carried out using the ChimeraX 1.10.1 software [46].

Additionally, MD simulations (100 ns) were performed to examine the possible effect of glycosylation on the interaction between the Lewis b tetrasaccharide and the BabA binding site. For glycosylation, System 3 was used as a reference, composed of complex-type N-glycans, and Le^b^ was positioned in its binding site. As a control system, a version of BabA with the antigen present but without glycosylation was constructed. All MD parameters were as described for the simulations without the Le^b^.

## 3. Results

### 3.1. Stability Analysis of Recombinant BabA Expressed in E. coli

**Figure 1:**
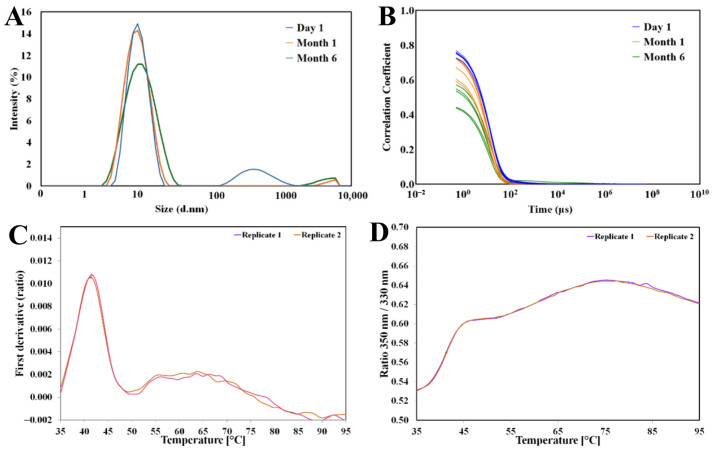
Recombinant BabA storage stability and thermostability assessment. (**A**) A plot of intensity versus size shows a single major peak and only a small proportion of aggregated BabA. (**B**) Correlogram of correlation coefficient that was used for cumulants analysis (see Table 1). BabA used for DLS was prepared at 0.5 mg/mL in PBS and stored at 4 °C. DLS measurements are shown at 1 day, 1 month, and 6 months after production of BabA sample. (**C**) shows the first derivative of the 350 nm/330 nm fluorescence ratio for BabA at a concentration of 0.5 mg/mL in PBS, with the peak at 39 °C indicating the melting point of the protein (replicate measurements are shown). (**D**) Shows the smoothed 350 nm/330 nm ratio fluorescent profile of the protein from 35 °C to 95 °C (replicate measurements are shown).

Dynamic light scattering (DLS) was performed on non-glycosylated recombinant BabA protein expressed in *E. coli* to investigate the thermal stability of the protein. The DLS showed a relatively similar mean z-average hydrodynamic diameter (*D_H_*) for the recombinant BabA protein when stored at 4 °C for up to 6 months; however, there was variation in the standard deviation (SD) at the 6-month time point (Table 1). The polydispersity index (PDI) and intensity versus size histograms indicate a relatively monodisperse population that was similar for all time points measured for the BabA protein, suggesting the protein is stable at 4 °C (Figure 1A). The correlation coefficients show a uniform shape indicating a similar major particle population, but a gradual decline in initial correlation coefficient values over time indicates that the BabA protein may have precipitated slightly after 6 months of storage (Figure 1B).

To assess the thermal stability of the BabA protein, nanoDSF was performed with a sample of BabA at 0.5 mg/mL to determine the melting point. The first derivative shows a low melting temperature of 39 °C (Figure 1C) in two replicates of the BabA protein. Furthermore, the smoothed fluorescence ratio (Figure 1D) shows a peak around 35 °C to 40 °C where the protein begins to unfold.

**Figure 2:**
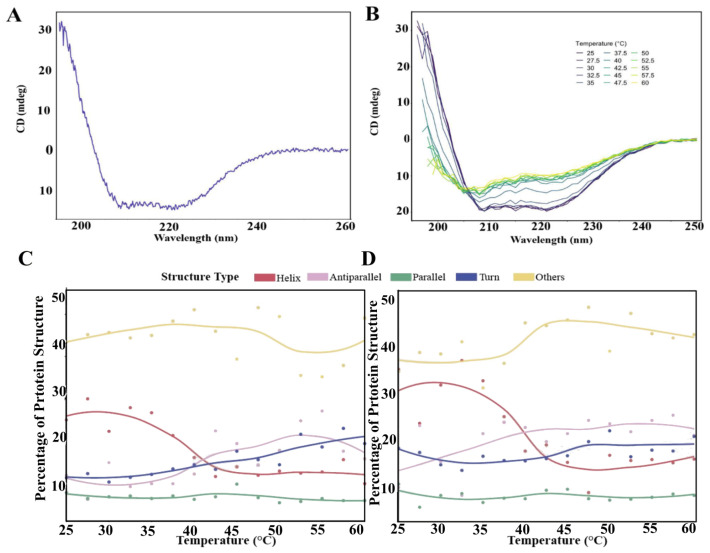
BabA secondary structure and temperature sensitivity by circular dichroism. (**A**) CD spectra obtained from a 0.15 mg/mL BabA solution in 0.5× PBS. (**B**) CD melting curve spectra, indicating a melting point of close to 40 °C. (**C**) Melting point CD scan secondary structure content vs. temperature at 195–250 nm range and (**D**) 200–250 nm range. Graphs track the change in secondary structure across the selected temperatures (helix, red; antiparallel, pink; parallel, green; turn, blue; others, yellow). Bioinformatic analysis was completed using the BeStSel online tool [29]. There is an obvious decline in helical content (red) across both ranges around the melting point of 40 °C, with many other elements remaining flat or marginally increasing.

Circular dichroism spectra were obtained for non-glycosylated recombinant BabA to elucidate the structural characteristics of the protein and further examine the melting point in vitro. The CD data of BabA obtained at 20 °C are characteristic of a mixed α-helix/β-sheet protein with a higher proportion of α-helix (Figure 2A). Across the temperature series, there are two obvious clusters of CD spectra, with a transition state occurring between 35 °C and 40 °C, with the 37.5 °C curve sitting right between the two clusters of lines (Figure 2B). This coincides with a sharp decline in the helical content, as observed in the secondary structure composition vs. temperature graphs, indicating a loss of secondary structure (Figure 2C,D). This similarly coincides with an increase in the “Others” content, which is very pronounced (Figure 2D). These results indicate that non-glycosylated BabA has low thermal stability and may not function optimally between 37.5 and 40 °C, where the protein is unfolding, which is remarkably close to the temperature *H. pylori* would encounter in the human gastric mucosa. While the periplasm is where disulfides are normally formed in *E. coli*, we note a possibility that disulfides were not present in the BabA used in this study. However, the residual structural content observed by CD at 60 °C (Figure 2B; a fully unfolded protein would have a CD minimum of 190–200 nm) does support the presence of disulfides in the BabA purified from the periplasm.

### 3.2. Sequence-Based Prediction of Glycosylation Sites of BabA

**Figure 3:**
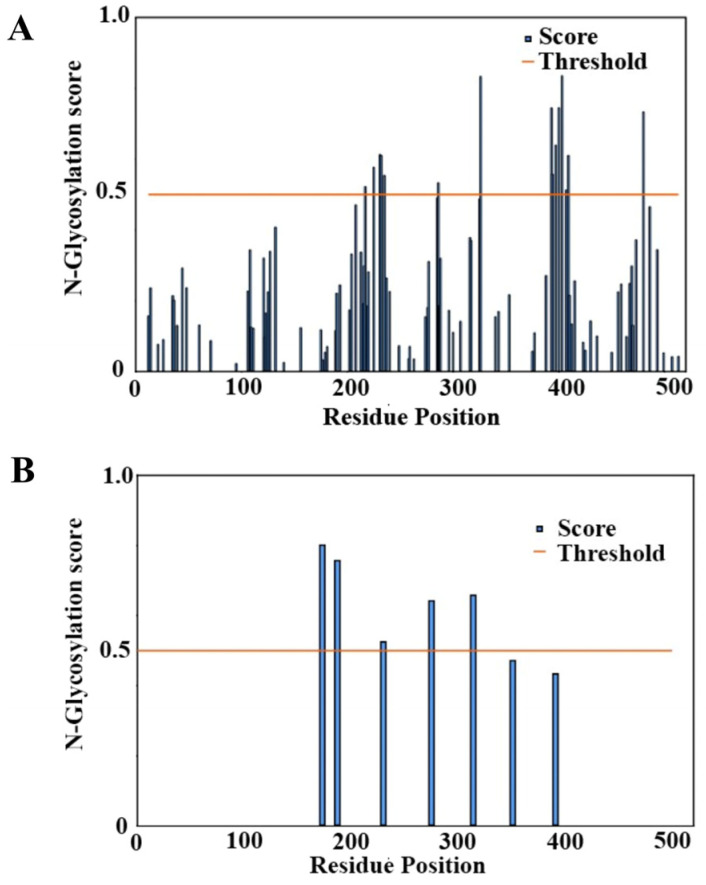
Predicted glycosylation for the BabA extracellular region. (**A**) O-glycosylation prediction of BabA. At a threshold of 0.5 for a predicted site, 14 potential sites for O glycosylation were observed over this threshold within the BabA extracellular region sequence using NetOGlyc 4.0. (**B**) N-glycosylation prediction of BabA. At a threshold of 0.5 for a predicted site, five out of seven potential sites were observed above the threshold for N-glycosylation within the BabA extracellular region sequence from NetNGlyc 1.0.

To explore the potential for O-glycosylation of BabA, the sequence of the recombinant protein was examined using a neural network prediction algorithm, NetOGlyc [31]. A total of 14 potential O-glycosylation sites were identified above the 0.5 threshold (Figure 3A). All the O-glycosylation sites identified were located between residues 217 and 480 (Table S2). While this range of residues is large and includes the head, handle, and crown domains, it does notably include the binding site in the crown domain β-sheet, which prominently features residues in the 190–240 range. NetNGlyc returned a lower number of potential glycosylation sites compared to NetOGlyc. NetNGlyc was developed using human proteins and examines the sequence context of Asn-Xaa-Ser/Thr sequons [30]. In the BabA protein, seven potential glycosylation sites were identified, and five of the sites were above the 0.5 score threshold (Figure 3B). Additionally, the glycosylation sites were located on residues Asn173, Asn187, Asn230, Asn275, and Asn314 (Table S2). Of these residues, Asn173, Asn187, Asn230, and Asn275 are all located in the β-Sheet of the crown domain of the protein, while Asn314 is in one of the α-helix structures within the head domain of the protein.

### 3.3. Structure-Based Prediction of Glycosylation of the BabA Extracellular Region

**Figure 4:**
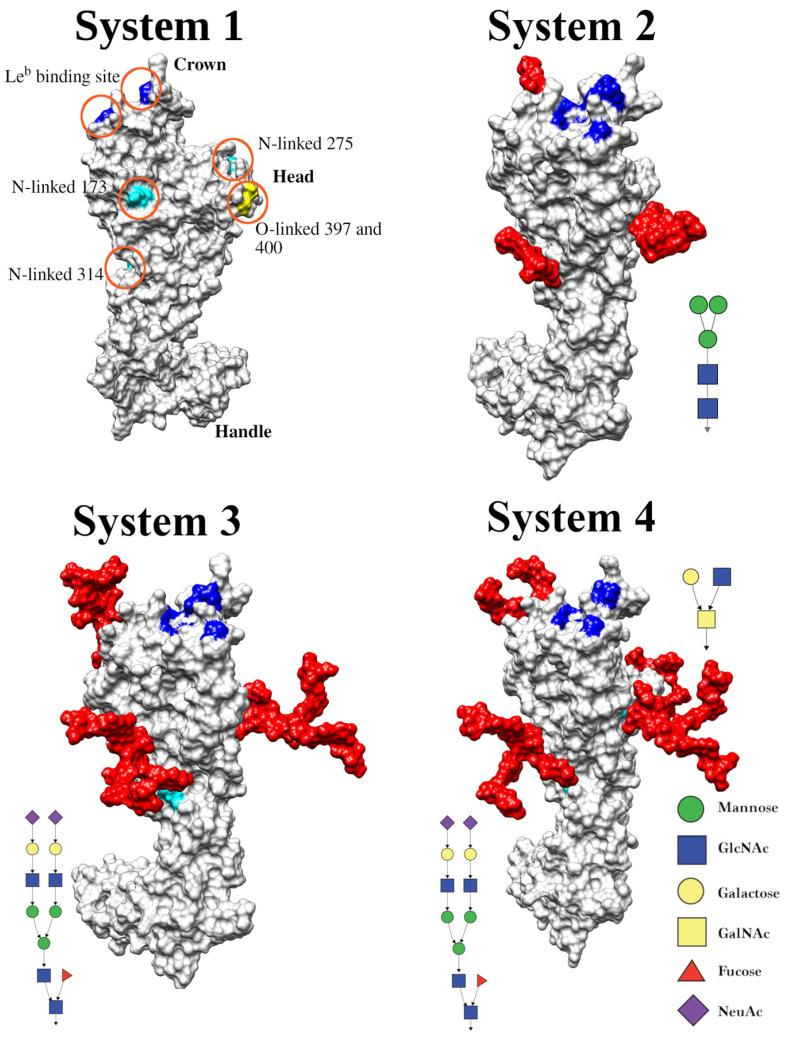
Structural representation of BabA glycosylation models. Glycosylation models of BabA (PDB: 4ZH7) at N-linked sites (173, 275, 314) and O-linked sites (397, 400). System 1: Non-glycosylated BabA structure, with the protein shown in grey. Predicted N-glycosylation sites are highlighted in cyan, and O-linked sites in yellow. System 2: BabA with Core N-linked glycans. System 3: BabA with Complex N-linked glycans. System 4: BabA with Complex N-linked glycans and core O-linked glycans. All glycans are shown in red, and the Le^b^ binding site is highlighted in blue in all panels.

GLYCAM-web utilizes the 3D structure of the protein and solvent-accessible surface area (SASA) scores to identify potential glycosylation sites and attach user-selected ligands to the solvent-accessible locations [33]. The top five most biologically likely (as determined by GLYCAM-web) glycosylation sites were selected and glycosylated by using the Core N-glycan (Figure 4). Additionally, a complex-type N-glycan was used at the N-linked sites, and a Core 2-type O-glycan was attached at the O-linked sites. The various glycans were assessed to evaluate the effect of characteristics such as their chemical structure and size. Of the five glycosylation sites selected, two were O-linked glycosylation sites and three were N-linked glycosylation sites (Table 2). The two O-glycosylation sites were relatively close together at residues Thr397 and Thr400 on a loop in the head domain of the protein. The N-glycosylation sites were further apart at Asn173, Asn275, and Asn314. (Figure 4). Lower-probability O-glycosylation and N-glycosylation sites were identified by GLYCAM-web but are not pictured here. Additionally, while more glycosylation sites were identified by NetNGlyc and NetOGlyc, the SASA analysis from GLYCAM-web did not support these (Table 2).

Overall, the analysis performed using GLYCAM-web identified several N-glycosylation and O-glycosylation sites on the outer membrane region of the BabA protein, the majority linked to the head domain of the protein and one N-glycosylation site in the crown domain of the protein. The highest-scoring site overall was a potential N-glycosylation site in the head domain. However, the N-glycosylation site located at Asn173, is potentially the most important due to its proximity to the crown domain of the protein, which includes the active site of the protein.

### 3.4. GlycoSHIELD Analysis

**Figure 5:**
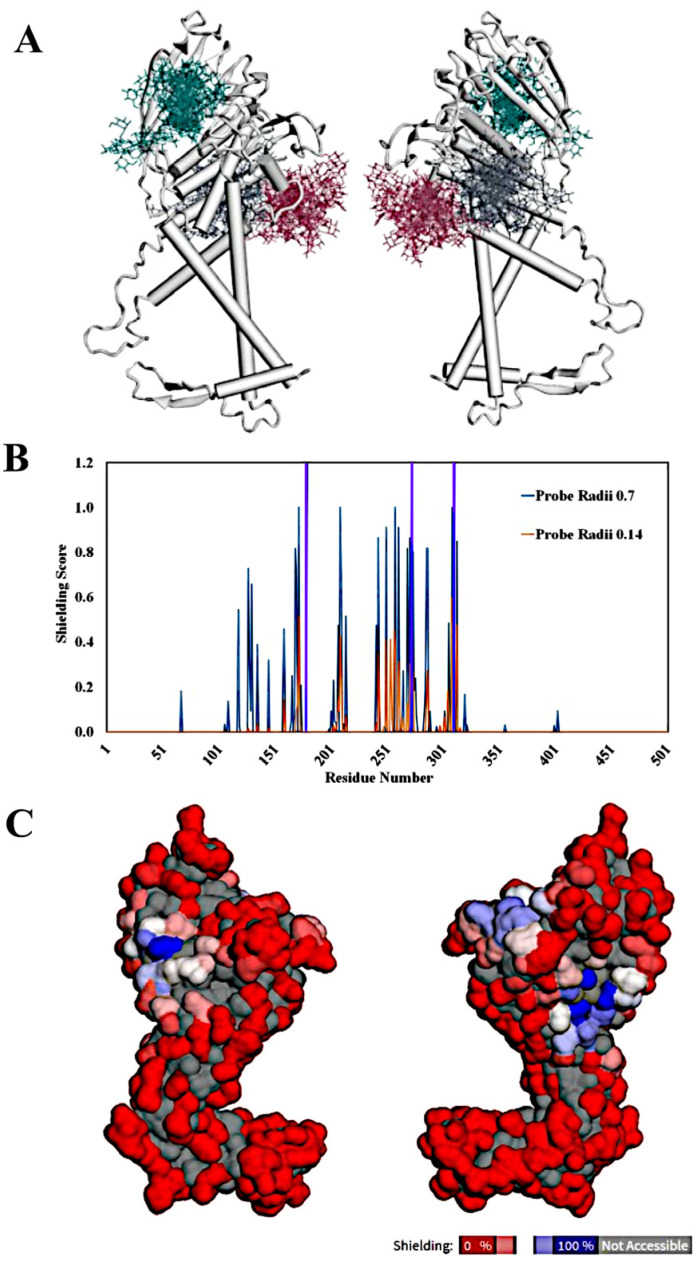
Predicted glycan shielding of BabA. (**A**) 3D structure of BabA with simulated N-glycosylation sites of the protein with attached Man3GlcNAc2 ligands. (Site N1 is displayed in green, site N2 is displayed in red, and site N3 is displayed in black). The N-glycans can be seen to potentially shield portions of the head and crown domains. The left and right images show the BabA protein rotated approximately 180° around its long axis. GlycoSHIELD [35] was used to attach the glycan structures to the 3D model of BabA (**B**) The BabA SASA shielding scores of the residues from GlycoSHIELD. The purple lines indicate the position in the sequence of the glycosylated Asn residues. (**C**) SASA 3D heat map of the shielding provided by the glycosylation of the protein at the 0.7 probe radii. The two protein positions shown are an approximate 180° mirror image to depict the whole protein.

The N-glycosylation sites were selected for further analysis due to their position in and around the head and crown regions of BabA. Notably, site N1 is close to the BabA-Le^b^ binding site in the crown β-sheet domain of the protein. Figure 5A displays the predicted ensembles for the attached carbohydrates on the BabA model. GlycoSHIELD aligns every glycan conformer based on the protein backbone of the Asn residues selected, flanked by a Thr/Ser, with a soft exclusion threshold of 0.7 Å. The glycan conformers show potential shielding surrounding the head and crown domains of the protein and around the binding site in the crown domain, with positions N2 and N3 covering a continuous area of the protein.

Related to the potential shielding effect of the glycans is the solvent-accessible surface area of the protein, which is reduced by the addition of N-glycans. Figure 5B shows the shielding score of the different residues on the protein. Residues surrounding the N-glycosylation sites show increased shielding scores, particularly using a 0.7 nm radius probe, where a score of one is no longer solvent accessible. A heatmap of the shielding effect of N-glycans is presented on the 3D structure of the BabA protein (Figure 5C). Large areas of the head and crown domains of BabA are shielded by the three N-glycans examined in this study. It is likely that a larger proportion of the surface of BabA would be shielded by the addition of O-glycans.

### 3.5. Molecular Dynamics Simulations of Glycosylated Versions and Non-Glycosylated BabA

**Figure 6:**
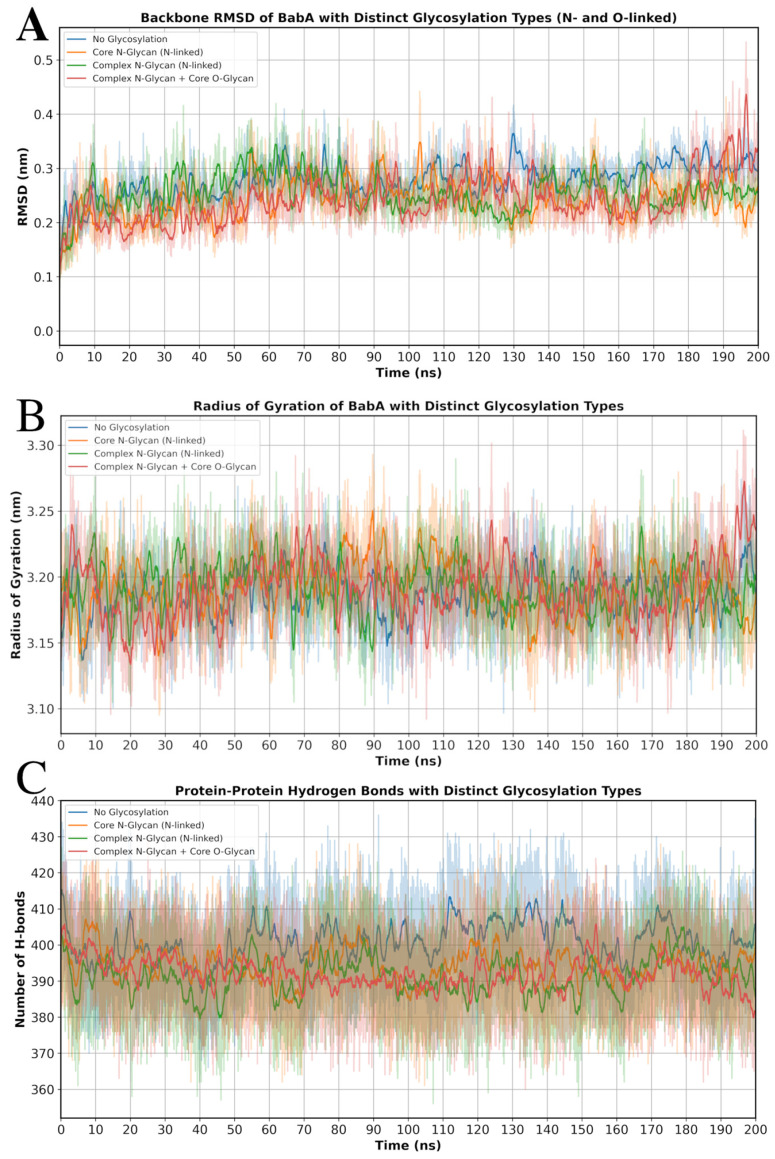
MD analysis of non-glycosylated and glycosylated BabA systems. (**A**) RMSD comparison based on protein backbone. (**B**) Radius of gyration comparison using whole protein structure. (**C**) Comparison of hydrogen bonds formed within protein structure across four systems. Production MD simulations were run for 200 ns.

Following GlycoSHIELD, molecular dynamics (MD) simulations were performed to examine the possible effect of glycosylation on the structural stability of BabA. Overall, the four systems (see Figure 4 and Table S1), including the non-glycosylated BabA and the three glycosylated variants, displayed similar values for the global structural indicators. There were no significant differences among the four systems when the root mean square deviation (RMSD) of protein backbone atoms, radius of gyration (Rg), and the number of hydrogen bonds (Hbonds) were all calculated over the entire protein structure (Figure 6 and Table 3).

**Figure 7:**
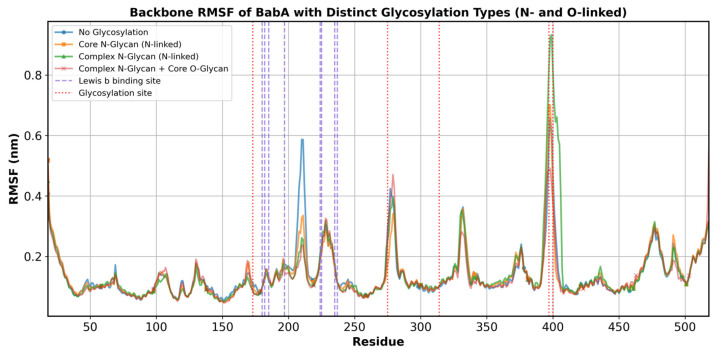
Root Mean Square Fluctuation (RMSF) per residue for the four BabA systems. The plot shows the RMSF of the protein backbone across all residues. The non-glycosylated BabA is represented by the blue solid line, the core N-glycosylated system (System 2) by the orange solid line, the complex N-glycosylated system (System 3) by the green solid line, and the complex N- and O-glycosylated system (System 4) by the red solid line. Red dotted lines indicate glycosylation sites (N173, N275, N314, T397, and T400), while purple dashed lines mark the residues involved in the Le^b^ binding site (C180, G182, N185, N197, D224, S225, S235, and T237). As the H1 glycan represents a trisaccharide portion of the Le^b^ tetrasaccharide determinant, only the Le^b^ binding residues were considered, as this should also account for the H1 binding site.

Since the global shape and mobility appeared similar between the non-glycosylated and glycosylated versions of BabA during the MD simulations, we next examined if there were any local effects of glycosylation on the protein structure. The root mean square fluctuation (RMSF) was examined for MD simulations of the four versions of BabA (Figure 7). Comparison of the RMSF for the four systems revealed that the glycosylation sites can exhibit changes in flexibility during the MD simulations. The N275, T397, and T400 sites were the most impacted by glycosylation, with a tendency to reduce mobility in the glycosylated systems. The region of BabA with the most differences in RMSF was between residues 209 and 211, which decreased in flexibility between 0.248 nm to 0.312 nm with glycosylation (Figure 7 and Table S3). This effect is seen further with increases in the size of the N-glycans and the addition of O-glycans.

**Figure 8:**
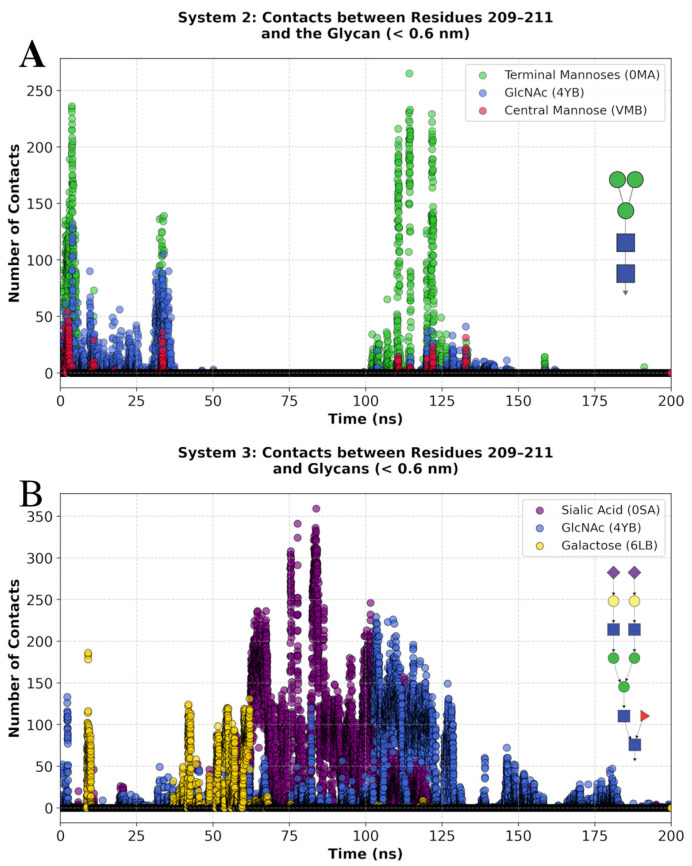
Time evolution of the number of contacts (<0.6 nm) between residues 209–211 and different glycan sections during the simulation. (**A**) System 2, showing contacts between the region and distinct glycan parts: terminal mannoses (0MA) in green, GlcNAc (4YB) in blue, and central mannose (VMB) in red. (**B**) System 3, showing contacts with the glycan segments: Sialic Acid (0SA) in purple, GlcNAc (4YB) in blue, and Galactose (6LB) in yellow.

To determine if differences in the flexibility of the 209–211 loop are directly influenced by glycosylation, the non-bonded contacts of residues within 0.6 nm of the glycans were monitored (Figure 8). Glycan-protein interactions occurred mainly during two segments of the MD simulation for System 2 (between 0 and 30 ns and 100 and 130 ns), whereas in System 3, they were clearly observed between 40 and 130 ns. These glycan–protein interactions suggest that the glycans can affect the local flexibility of specific protein regions at different time intervals, with both the terminal portions and the core structure of the glycans engaging in interactions with the protein.

**Figure 9:**
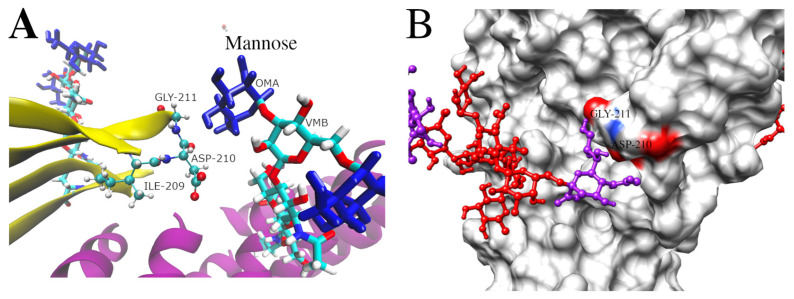
Visualization of the interaction between residues 209–211 and glycans. (**A**) Representation of the interaction between terminal mannoses (blue) and the 209–211 region in System 2. (**B**) Representation of the interaction between sialic acid (purple) and the 209–211 region in System 3.

To dissect which parts of the glycans are involved in the interaction with residues 209–211, the contacts for System 2 were segmented into the terminal mannoses (0MA), the two GlcNAc residues (4YB), and the central mannose (VMB) (Figure 8A and Table S4). This analysis showed the terminal mannoses (0MA) to participate in most of the interactions (73,973 contacts) with the 209–211 loop over 16 ns of simulation time. The two GlcNAc (4YB) residues participated in 35,477 contacts over 28 ns (Figure 8A and Table S4). In the case of System 3, the sialic acid residues (0SA) were involved in 430,196 contact occurrences over 62 ns of simulation time. The GlcNAc residues (4YB) participated in 263,620 contact occurrences over 72 ns (Figure 8B and Table S4). Taken together, these data illustrate how a glycan can impact the flexibility of a protein region by participating in dynamic interactions of various durations, which are influenced by the type and length of the glycan chain. The approach to the 209–211 loop of the terminal mannose of the Core N-glycan (System 2) and a sialic acid residue of the Complex N-glycan (System 3) are illustrated in Figure 9. This supports the idea that the glycan size directly influences the mobility of nearby protein regions.

Since the complex N-glycans displayed some interactions with the BabA glycan binding site, we assessed if glycosylation affected the stability of the Lewis b interaction with BabA using MD simulations. Analysis of the RMSD and RMSF showed no significant differences between the non-glycosylated and glycosylated systems in the presence of the Le^b^ tetrasaccharide (Figure S2). The RMSD of the Le^b^ ligand showed slightly higher stability in the presence of N-glycans; therefore, the contacts between the terminal regions of the complex-type glycan and Le^b^ were evaluated. The results confirmed the presence of interactions for approximately 25 ns of the simulation (cutoff of 0.6 nm) suggesting that glycosylation may promote reduced flexibility in the Le^b^ ligand. Although the differences in Le^b^ stability are minor, they point to the need for further investigation into BabA glycosylation and its role in recognition of Le^b^ and related glycans.

## 4. Discussion

Glycosylation has previously been demonstrated to have several effects on protein structure and function. One example of such an effect occurs with branched glycans of appropriate hydrophobicity conferring enhanced folding, stability, and activity, while glycans of lower hydrophobicity can hinder folding while still increasing stability [47]. This study examined the stability of the *H. pylori* outer membrane protein BabA after expression in *E. coli* BL21. Dynamic light scattering yielded comparable results for the protein when stored at 4 °C, when evaluated at day one, month one, and at six months post-purification. Thermal stability studies showed that the recombinant BabA had an unexpectedly low melting temperature of 39 °C, which was supported by CD measurements showing an unfolding of BabA that commenced around 32.5 °C and concluded at 40 °C. This observation prompted a series of computational studies to examine how glycosylation may affect the stability of the BabA protein. NetNGlyc [30] and NetOGlyc [31,32] revealed multiple potential glycosylation sites based on the BabA sequence, and analysis with GLYCAM-web [33,34] found three biologically probable N-glycosylation sites and three O-glycosylation sites.

We examined the potential for the N-glycans to shield parts of the protein surface to determine the effect glycosylation has on the surface availability of protein residues. GlycoSHIELD [35] demonstrated a significant reduction in the solvent-accessible surface area of the protein surrounding the glycosylation sites, and MD studies demonstrated a potential stabilizing effect on the protein at the points of glycosylation. These results support the notion that production of recombinant BabA could benefit from utilizing systems that glycosylate the protein to improve stability and reduce SASA of the BabA polypeptide chain.

Other studies have examined the effect of N-glycosylation on protein stability with comparable results. For example, Lee et al. [24], explored the stability of six glycoproteins compared to the non-glycosylated versions using MD simulations, finding decreases in RMSF and RMSD for all six proteins, theorizing that glycosylation functioned as a ‘molecular glue’, holding residues in place due to the favorable interactions provided by the covalently bound glycans [24]. An earlier study conducted a statistical analysis into the effects of post-translational modifications (PTM) on proteins with PDB structures, which also indicated reductions in RMSD and increases in stability, most notably from phosphorylation but also from glycosylation. Significant changes to protein structural conformation were also identified [48]. Studies focusing on SARS-CoV-2 glycoproteins have identified several roles for glycosylation, including demonstrating epitope shielding, regulation, and activation of lectin pathways [49]. Specifically, N-glycans attached to the S protein have been found to contribute to conformational plasticity, structural stability, and receptor binding [50,51,52,53]. In the case of BabA, glycosylation did not show a significant effect on the global stability of the protein, as indicated by indicators such as RMSD, radius of gyration, and hydrogen bonds. However, local changes were observed both at the glycosylation sites and at the residues interacting with the glycans, with a tendency to reduce residue mobility, as shown by local indicators such as RMSF and contact analysis. Furthermore, the importance of glycan size and type was confirmed, as their specific characteristics influenced the flexibility of the interacting residues, as well as the duration and number of contacts formed. In System 3, a slight interaction was observed between the complex N-glycan and the Le^b^ binding site. The presence of N-glycans was associated with a minor increase in stability of the Le^b^ tetrasaccharide within the BabA binding site, which opens the possibility for future studies exploring the effects of glycosylation on the Le^b^ binding site and its ligand.

Glycan shielding is particularly relevant in drug design when using protein 3D structures to identify specific targets, as many surface proteins are glycosylated and glycans can form extensive networks that cover large portions of a glycoprotein’s surface, altering protein conformation and drug interactions. GlycoSHIELD was developed with that application in mind and was validated against N-cadherin and SARS-CoV-2 S protein, in comparison to MD simulations and cryo-EM maps. This application demonstrates the significant diversity of glycan conformational profiles and shows the significant surface area that may be shielded by glycans [35]. Due to the significant conformational flexibility of N-glycans, the impact of glycan shielding on protein structure and function requires further consideration in future studies [54]. This current study identifies a strong role for the shielding of protein structures by complex glycosylation in the *H. pylori* protein BabA and is supported by changes in the structural behavior of the protein under different glycosylation conditions.

Less is known about bacterial glycosylation in comparison to eukaryotic glycosylation pathways, and the number of bacterial proteins that have had structural effects of glycosylation explored is relatively limited. To date, *C. jejuni* is the best-characterized bacteria in terms of glycosylation and was the first identified to be capable of complex glycosylation [3,18,20,55]. Furthermore, previous studies have demonstrated difficulties in experimentally studying bacterial glycoproteins, as establishing robust glycosylation systems in *E. coli* is a challenge [56,57,58,59]. The exolytic lytic transglycosylase, Cj0843, of *C. jejuni* has been observed to possess at least five N-glycosylation sites, of which four have been mapped by mass spectrometry. However, the functional importance of these sites has not yet been defined other than the likelihood that they increase bacterial fitness [60]. A more recent study examining *C. jejuni* efflux pump CmeA focused on accurately producing an N-glycosylated form of the protein using an extensively validated glycocompetent *E. coli* expression system. The study demonstrated that the glycosylation of the protein modulated the global structure of the protein and modulated protein–protein interactions, which in turn enhanced protein thermostability [61]. Like the local reductions in RMSF that we saw at glycosylation sites near the active site of BabA, another study examined the effect that ligand complexes have on the bacterial enzyme MurA, an important protein in bacterial cell wall synthesis [32]. It found that the glycan UDP-N-acetylglucosamine interacted tightly with the MurA active site, stabilizing a closed conformation of the protein and significantly dropping the RMSF of the residues involved in the interaction during MD simulation [62].

Further exploration of the effect of glycosylation on bacterial proteins, along with further development of expression systems that accurately produce glycosylated bacterially derived proteins, could lead to significant improvements in drug development and research into the structure and function of these glycoproteins.

## 5. Conclusions

*H. pylori* is known to perform complex glycosylation and employs a generalized glycosylation system to form both the bacterial LPS and to glycosylate outer membrane proteins (OMP). This study identified several potential glycosylation sites in both the crown and head domains of the BabA protein and was able to simulate the effect of glycosylation on local regions of the protein, such as the glycosylation sites and the areas of glycan interaction, indicating increased local stability. It also highlighted the importance of glycan characteristics, such as size and composition. This illustrates the importance of glycosylation in protein stability, suggesting that expression of BabA and similar proteins should consider the impact of glycosylation. This study used glycans and glycosylation typically expressed in mammalian systems, indicating that mammalian cells or systems with similar glycosylation mechanisms may be better suited to the expression of proteins such as BabA rather than standard *E. coli* expression systems. Future studies could involve the production of BabA in expression systems capable of complex glycosylation to produce a more biologically stable protein for research and drug development.

## Figures and Tables

**Table 1:** Dynamic light scattering of recombinant BabA.

Month	Mean *D_H_* ^a^ (nm)	SD ^b^ *D_H_* ^a^ (nm)	Mean PDI ^c^	SD ^b^ PDI ^c^
0	9.58	0.27	0.3	0.02
1	8.17	0.32	0.19	0.04
6	9.11	1.9	0.18	0.04

^a^ Hydrodynamic diameter (*D_H_*), ^b^ standard deviation (SD), ^c^ polydispersity Index (PDI).

**Table 2:** Glycosylation sites identified by GLYCAM-web.

Context	Chain	Linkage	Residue Number	SASA ^a^
LVN * QT	A	N	314	84.1 +
ASN * SS	A	N	275	82.8 +
AGT * GG	A	O	397	76.5 +
KVN * VT	A	N	173	70.5 +
GGT * QG	A	O	400	40.4 +
PGT * VT	A	O	407	38.2

^a^ Solvent accessible surface area, * indicates the residue that was glycosylated, + indicates a SASA score of above the cutoff of 40 for prediction.

**Table 3:** Average values and standard deviations of key structural indicators of non-glycosylated and glycosylated BabA systems.

Indicator	System 1 ^a^	System 2	System 3	System 4
RMSD (Backbone) [nm]	0.243 ± 0.038	0.244 ± 0.044	0.254 ± 0.039	0.241 ± 0.049
Radius of Gyration [nm]	3.186 ± 0.024	3.192 ± 0.026	3.192 ± 0.025	3.191 ± 0.029
H-bonds (average)	400.64 ± 9.45	394.25 ± 8.87	391.2 ± 9.2	392.1 ± 8.7

^a^ System 1—non-glycosylated BabA model (control), System 2—BabA model glycosylated with Core N-glycans, System 3—BabA model glycosylated with Complex N-glycans, System 4—same as System 3, with the addition of Core 2 O-glycans.

## Data Availability

All data is presented in this paper and Supporting Information are available from the authors on request.

## Supplementary Materials

The supporting information can be downloaded at https://www.mdpi.com/article/10.3390/biom15101480/s1. Table S1: Summary of BabA glycosylation systems, Table S2: Predicted BabA glycosylation sites, Table S3: Comparison of RMSF values for glycosylated and non-glycosylated protein systems, Table S4: Summary of contact counts and simulation time with any contact between residues 209−211 and N-glycans in System 2 (core N-glycan) and System 3 (complex N-glycan), Figure S1: Recombinant BabA purified from the periplasm of *E. coli,* Figure S2: Impact of N-glycosylation on the BabA-Lewis b complex assessed by a 100 ns molecular dynamics simulation. Supplementary Material S2: Original Western blot images of Figure S1A,B.

## Abbreviations

The following abbreviations are used in this manuscript

BabA	blood group binding adhesin
CD	circular dichroism
CV	column volume
D_H_	hydrodynamic diameter
DLS	dynamic light scattering
IMAC	immobilized metal affinity chromatography
IPTG	isopropyl β-D-1-thiogalactopyranoside
Le^b^	Lewis b
LPS	lipopolysaccharide
MD	molecular dynamics
nanoDSF	nano differential scanning fluorometry
OD	optical density
OMP	outer membrane proteins
PDI	polydispersity index
PTM	post-translational modifications
R_g_	radius of gyration
RMSD	root mean square deviation
RMSF	root mean square fluctuation
SASA	solvent accessible surface area
SD	standard deviation
