# Supplementary material for: Predicting the Impact of Glycosylation on the Structure and Thermostability of Helicobacter pylori Blood Group Binding Adhesin
Source: Biomolecules. 2025 Oct 21;15(10):1480. doi: 10.3390/biom15101480 (PMC12563011; doi:10.3390/biom15101480)
Supplement: Supplementary file 1 [file biomolecules-15-01480-s001.zip › biomolecules-3876591-supplementary-2.pdf]

Predicting the impact of glycosylation on the structure and thermostability of *Helicobacter pylori* blood group binding adhesin

Daniel Sijmons[1,2#], Heber Islas Rios[1#], Benjamin R. Turner[1], Emma Wanicek[1], Jessica K. Holien[1], Anna K. Walduck[1,3], Paul A. Ramsland[1,4,5*]

[1]School of Science, RMIT University, Melbourne, VIC 3000, Australia.
[2]Department of Microbiology and Immunology, Peter Doherty Institute for Infection and Immunity, University of Melbourne, Melbourne, VIC 3000, Australia.
[3]Rural Health Research Institute, Charles Sturt University, Orange, NSW 2800, Australia
[4]Department of Immunology, Monash University, Melbourne, VIC 3004, Australia
[5]Department of Surgery, Austin Health, The University of Melbourne, Heidelberg, VIC 3084, Australia

#Equal Contribution

*Corresponding author: paul.ramsland@rmit.edu.au

Supporting Information

Table S1. Summary of BabA glycosylation systems

System	Description	Glycosylation Sites	Glycan Type (Name)
**System 1**	Non-glycosylated BabA model (control)	None	None
**System 2**	BabA model glycosylated with core N-glycans	Asn173, Asn275, Asn314	Manα1-3(Manα1-6)Manβ1-4GlcNAcβ1-4GlcNAcβ1–Asn
**System 3**	BabA model glycosylated with complex N-glycans	Asn173, Asn275, Asn314	Neu5Acα2-6Galβ1-4GlcNAcβ1-2Manα1-3(Neu5Acα2-6Galβ1-4GlcNAcβ1-2Manα1-6)Manβ1-4GlcNAcβ1-4(Fucα1-6)GlcNAcβ1–Asn
**System 4**	Same as System 3 with the addition of O-glycans	N-glycans: Asn173, Asn275, Asn314<br>O-glycans: Thr397, Thr400	Complex N-glycan + Core type 2: Galβ1-3(GlcNAcβ1-6)GalNAcα1-Thr

Table S2. Predicted BabA Glycosylation Sites

O-Glycosylation

Residues	Location	Score
TISS	217	0.52
SRAD	225	0.58
TTGV	231	0.61
TGVS	232	0.61
SYTE	235	0.55
TTTG	286	0.53
TPVG	326	0.83
STAG	393	0.75
TAGT	394	0.56
TGGT	397	0.64
TQGS	400	0.75
SAPG	403	0.84
TVTT	407	0.51
TTQT	409	0.61
SLQN	480	0.73

N-Glycosylation

Residues	Location	Score
NVTY	163	0.80
NCSS	177	0.76
NTTG	220	0.53
NSSE	265	0.65
NQTS	304	0.66
NASA	341	0.48
NPST	381	0.44

Table S3. Comparison of RMSF values for glycosylated and non-glycosylated protein systems.

Residue	System 1	System 2	System 3	System 4	Average RMSF Difference (nm)
210	0.587	0.328	0.261	0.238	0.312
211	0.586	0.335	0.255	0.237	0.31
209	0.475	0.277	0.216	0.188	0.248
212	0.438	0.266	0.195	0.193	0.22
208	0.43	0.283	0.214	0.184	0.203
403	0.218	0.129	0.661	0.167	0.194
402	0.283	0.159	0.645	0.23	0.18
399	0.614	0.573	0.933	0.438	0.179
404	0.153	0.12	0.588	0.13	0.164
398	0.657	0.7	0.926	0.49	0.16

*System 1 - Non-glycosylated BabA model (control), System 2 - BabA model glycosylated with core N-glycans, System 3 - BabA model glycosylated with complex N-glycans, System 4 - Same as System 3 with the addition of O-glycans.

Table S4. Summary of contact counts and simulation time with any contact between residues 209–211 and N-glycans in System 2 (Core N-glycan) and System 3 (Complex N-glycan)

System	Glycan unit	Contact count	Simulation time with any contact (ns)
**System 2**	Terminal Mannoses (0MA)	73973	16
	GlcNAc (4YB)	35477	28
	Central Mannose (VMB)	3921	4
**System 2 total**		**113371**	**48**
**System 3**	Sialic Acid (0SA)	430196	62
	GlcNAc (4YB)	263620	72
	Galactose (6LB)	62212	17
**System 3 total**		**756028**	**151**

* System 2 - BabA model glycosylated with core N-glycans, System 3 - BabA model glycosylated with complex N-glycans.

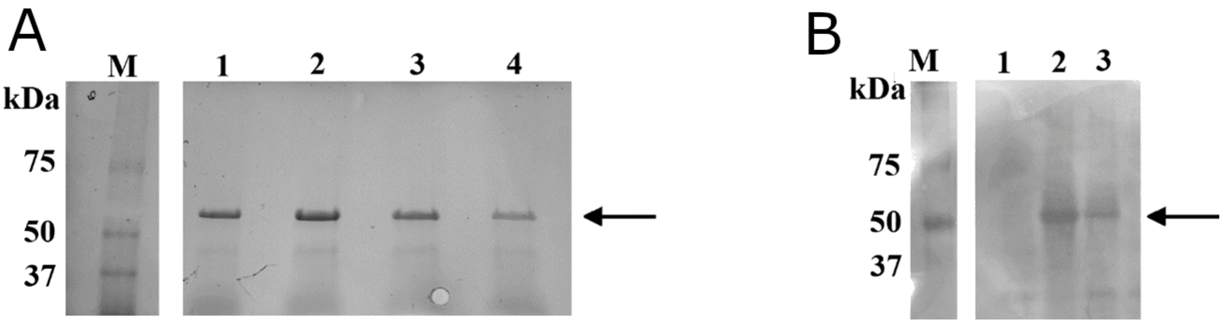

Figure S1. Recombinant BabA purified from the periplasm of *E. coli*. (**A**) Coomassie stained SDS-PAGE of purified recombinant BabA protein samples, which were all expressed at 16°C for 16 hours and purified by immobilised metal affinity chromatography (IMAC) (Lanes 1-4). (**B**) Western blot of purified recombinant BabA protein, Lane 1: negative control, Lane 2-3: HIS-tag labelled recombinant BabA. Densitometry of the BabA bands using ImageJ software provided peak volumes (area x intensity) of 92393.9 (Lane 2) and 22138.5 (Lane 3).

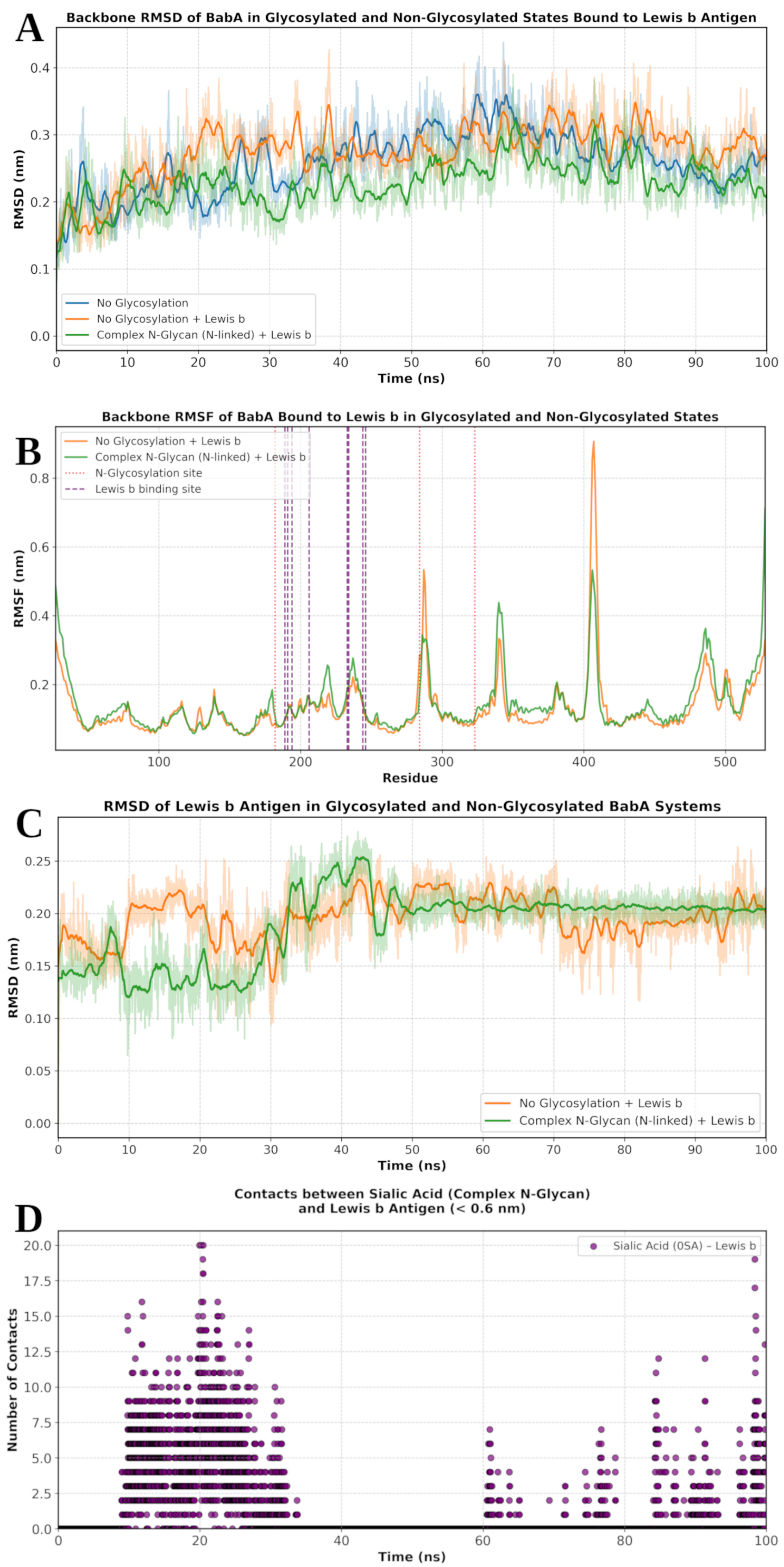

Figure S2. Impact of N-Glycosylation on the BabA–Lewis b complex assessed by a 100 ns molecular dynamics simulation. (A) Backbone RMSD of BabA for three systems: non-glycosylated BabA (blue), non-glycosylated BabA bound to Lewis b (orange), and glycosylated BabA with the complex N-glycans bound to Lewis b (green). **(B)** Backbone RMSF profiles of BabA show the comparison between non-glycosylated BabA bound to Lewis b (orange) and glycosylated BabA with the complex N-glycan bound to Lewis b (green). Red dotted lines indicate N-glycosylation sites, and purple dashed lines indicate Lewis b binding regions. **(C)** RMSD of the Lewis b antigen when bound to glycosylated and non-glycosylated BabA. **(D)** Number of contacts (< 0.6 nm) between the sialic acid of the complex N-glycan and the Lewis b antigen throughout the simulation.
