# Supplementary figures and images for: Predicting the Impact of Glycosylation on the Structure and Thermostability of Helicobacter pylori Blood Group Binding Adhesin
Source: Biomolecules. 2025 Oct 21;15(10):1480. doi: 10.3390/biom15101480 (PMC12563011; doi:10.3390/biom15101480)

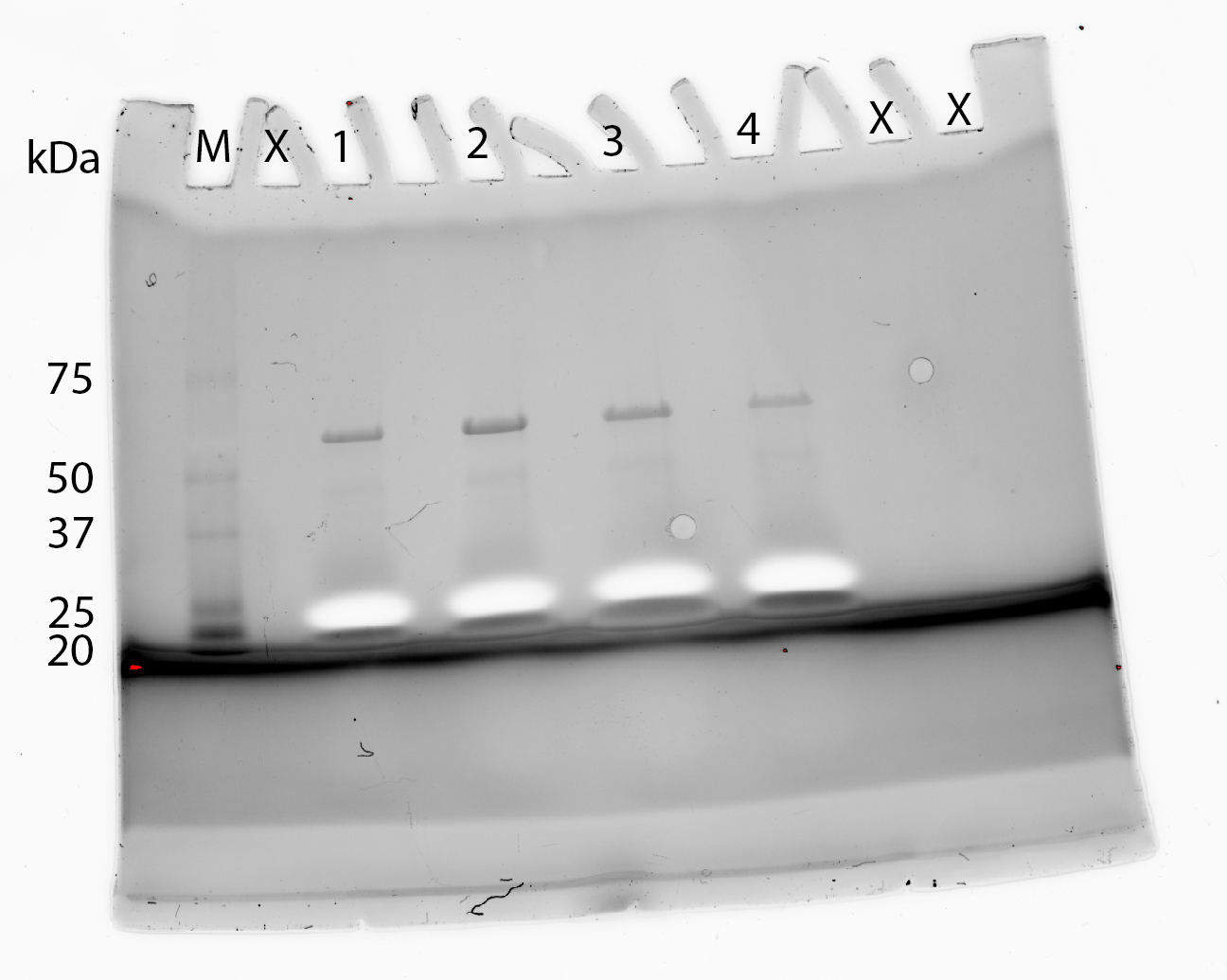

Supplement: Supplementary file 1 [file biomolecules-15-01480-s001.zip › OriginalGelBlot_FigureS1/FigureS1A_SDS-PAGE_Original_Annotated.png]

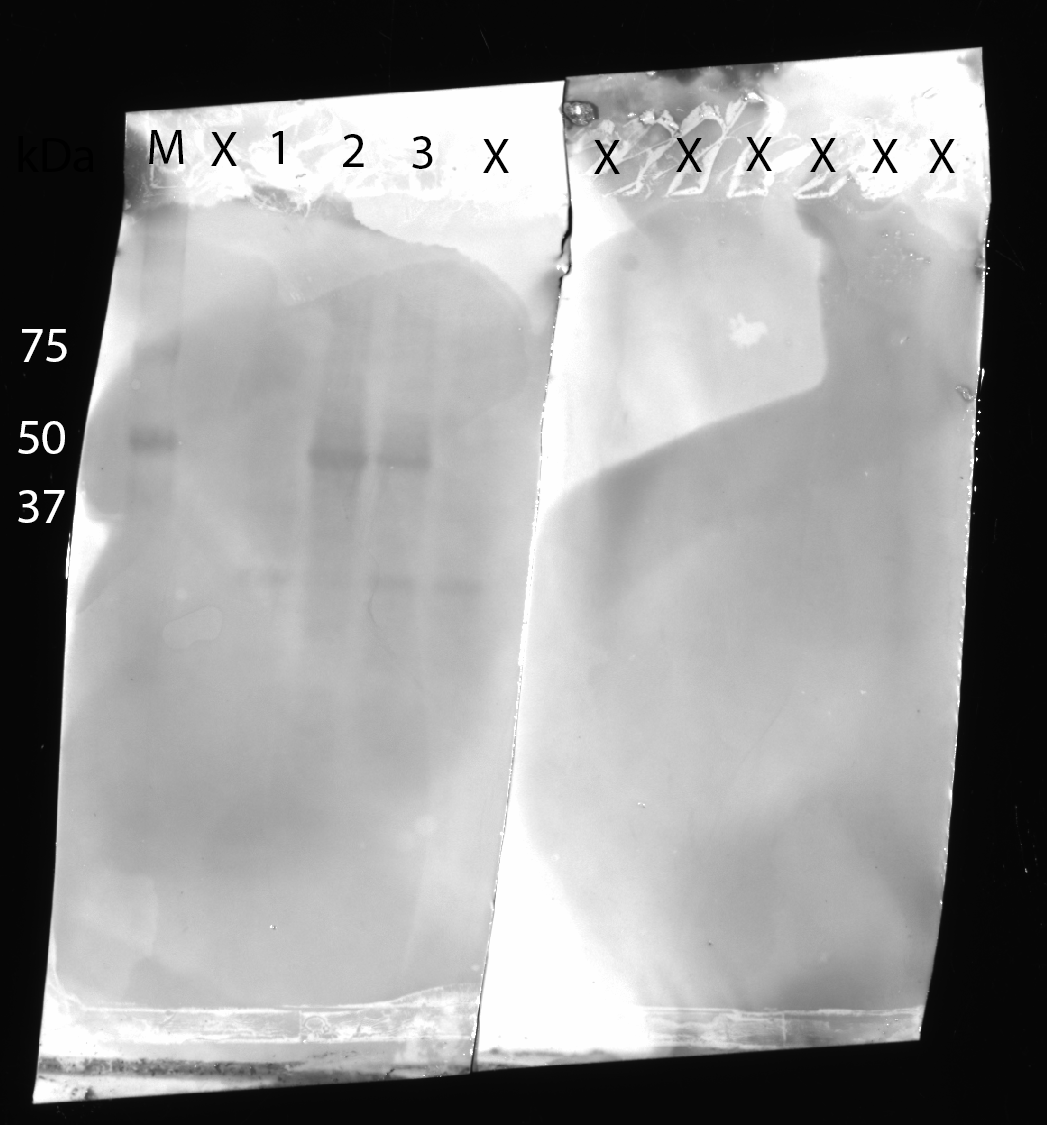

Supplement: Supplementary file 1 [file biomolecules-15-01480-s001.zip › OriginalGelBlot_FigureS1/FigureS1B_WestermBlot_Original_Annotated.png]
